# Activation of PPARγ by baicalin attenuates pulmonary hypertension in an infant rat model by suppressing HMGB1/RAGE signaling

**DOI:** 10.1002/2211-5463.12180

**Published:** 2017-03-08

**Authors:** Zhenjie Chen, Qiuxia Wang

**Affiliations:** ^1^ Department of PICUThe Children's HospitalZhejiang University School of MedicineHangzhouChina

**Keywords:** baicalin, infants, inflammation, PPARγ, pulmonary hypertension

## Abstract

Pulmonary hypertension (PH) is a vascular disease, and proinflammatory factors are strongly implicated in its pathogenesis, causing right ventricular (RV) hypertrophy and heart failure. Baicalin exhibits potent anti‐inflammation activity. This study aimed to investigate the curative effects of baicalin in an infant rodent model of PH and to further explore the underlying mechanisms. A PH model in infant rats was induced by hypoxia and the resulting rats were administered baicalin in incremental dosages. Invasive hemodynamic methods were used to measure mean pulmonary arterial pressure (mPAP) and RV end‐diastolic pressure (RVEDP). RV hypertrophy was assessed by mass pathology and histology. ELISAs were used to determine concentrations of high‐mobility group box 1 (HMGB1), secretory receptor for advanced glycation end products (sRAGE), interleukin 6 (IL6) and transforming growth factor β (TGFβ1) in bronchoalveolar lavage fluid (BALF). Electrophoretic mobility shift and phosphorylation in nuclear extracts were used to evaluate the activation of peroxisome proliferator‐activated receptor γ (PPARγ). Western blotting was used to detect the expression levels of heme oxygenase 1 (HO1), HMGB1, RAGE, IL6 and TGFβ1 in lung tissue. Baicalin administration significantly attenuated mPAP, RVEDP and RV hypertrophy in infant rats with PH. HMGB1, sRAGE, IL6 and TGFβ1 levels in BALF were also reduced by baicalin treatment. Baicalin activated PPARγ, which promoted expression of HO1. Furthermore, expression levels of HMGB1, RAGE, IL6 and TGFβ1 in lung tissue were dramatically decreased by baicalin in a dosage‐dependent manner. Baicalin showed curative effects in infant rats with PH. Activation of PPARγ that inhibited HMGB1/RAGE inflammatory signaling was involved.

**Abbreviations**
BALF: bronchoalveolar lavage fluid
CTGF: connective tissue growth factor
GAPDH: glyceraldehyde 3‐phosphate dehydrogenase
HMGB1: high‐mobility group box 1
HO1: heme oxygenase 1
IL6: interleukin 6
PH: pulmonary hypertension
PPARγ: peroxisome proliferator‐activated receptor γ
sRAGE: secretory receptor for advanced glycation end products
RV: right ventricular
TGFβ1: transforming growth factor β1

Pulmonary hypertension (PH) is characterized by elevated pulmonary vascular pressure and resistance. PH is now recognized as one of the pulmonary vascular diseases responsible for mortality in the susceptible population including infants. The phenotypes of PH in infants can be divided into four subtypes: (a) congenital heart disease related (infants often with left–right shunt congenital heart diseases; (b) acute or chronic hypoxia related; (c) neonatal developmental defect related; and (d) idiopathic forms 1, 2. Remodeling of the small pulmonary arteries is the leading manifestation of the pathology of PH 3, and it was reported that airway inflammation played important roles in this process, contributing to the development of PH 4. However, the exact mechanism is still not clear.

Secreted by activated monocytes and macrophages, high‐mobility group box 1 (HMGB1) is recognized as a critical mediator inducing inflammation 5. Under several pathological conditions, HMGB1 is released from nuclei and binds to receptor for advanced glycation end products (RAGE) 6. The activation of HMGB1/RAGE signaling would further activate mitogen‐activated protein kinase and nuclear factor‐κB signaling to initiate the production of many proinflammatory mediators such as transforming growth factor β1 (TGFβ1) and interleukins 7, 8. According to several previous studies, serum RAGE level was found to be dramatically increased in PH patients 9; expression level of HMGB1 in lung tissue from a PH animal model was also found to be increased significantly 10. These results indicated that HMGB1/RAGE signaling was involved in PH. The expression of peroxisome proliferator‐activated receptor γ (PPARγ) was found in both infiltrated immunocells and pulmonary structural cells. Both *in vivo* and *in vitro* experiments proved the anti‐inflammatory effect of PPARγ activation 11. More importantly, a study concerning acute lung injury indicated that PPARγ signaling activation would suppress HMGB1/RAGE signaling by inducing heme oxygenase 1 (HO1) 12.

Baicalin is one of the effective flavonoid constituents isolated from the Chinese medicinal herb huang qin (*Scutellaria baicalensis* Georgi). Baicalin exerts a variety of biological activities including cardioprotective, anticancer, antiproliferative, antioxidant and anti‐inflammatory effects 13, 14, 15. A few previous investigations found that baicalin exhibited therapeutic effects in an animal model of PH 16. However, the mechanisms are still not clear. In inflammatory animal models, baicalin was proved to decrease the level of HMGB1 17. Moreover, baicalin was found to show pharmacological activity as a PPARγ activator 18. Thus, it is reasonable for us to raise the hypothesis that baicalin could activate PPARγ/HO1 signaling, which ameliorates airway inflammation by inhibiting HMGB1/RAGE signal transduction.

In this study, we examined the effects of baicalin on hypoxia‐induced PH and the involvement of PPARγ/HMGB1/RAGE signaling in infant rats. This will provide not only knowledge of therapeutic molecular targets, but also a theoretical basis for clinical application of baicalin or baicalin‐containing compounds for treatment in infant PH.

## Materials and methods

### Animal study

The animal model protocol of PH infant rats was carried out in accordance with a previous study 3. Fifteen days after birth, female Wistar rats with their neonatal pups were divided into five groups evenly and randomly. These were a control group (Ctrl), a PH group (PH) and three baicalin‐treated groups (BT1–BT3). The treatment details for each group are listed in Table 1. The animal investigation protocols were carried out in accordance with the Guide for the Care and Use of Laboratory Animals (NIH Publication No. 85‐23, revised 1996). The protocols were approved by the Committee of Animal Use and Ethics in Children's Hospital Affiliated to School of Medicine of Zhejiang University (Sn. AC0312016).

**Table 1:** Animal groups and treatment

	Modeling	Treatment
Ctrl	Ambient room air for 3 weeks	Standard rat chow
PH	Hypobaric chamber (50 kPa) for 3 weeks	Standard rat chow
BT1	Hypobaric chamber (50 kPa) for 3 weeks	Oral administration of baicalin at dosage of 10 mg·kg^−1^·day^−1^
BT2	Hypobaric chamber (50 kPa) for 3 weeks	Oral administration of baicalin at dosage of 20 mg·kg^−1^·day^−1^
BT3	Hypobaric chamber (50 kPa) for 3 weeks	Oral administration of baicalin at dosage of 30 mg·kg^−1^·day^−1^

### Mean pulmonary artery pressure, right ventricular hypertrophy and right ventricular pressure measurements

The rats were anaesthetized by intraperitoneal injection of chloral hydrate (10% v/v, 0.3 mL·kg^−1^). The right external jugular vein was exposed and a polyethylene catheter (Millar Inc., Houston, TX, USA) with internal diameter 0.9 mm was inserted into the right ventricular (RV) or pulmonary artery. The catheter was connected with a pressure transducer (Millar) to record the right ventricular end‐diastolic pressure (RVEDP) and mean pulmonary artery pressure (mPAP). The heart samples were harvested and the RV tissue was separated from the left ventricle and interventricular septum. RV hypertrophy was indexed by the mass ratio of the right ventricle to the left ventricle plus septum (RV/LV+S).

### Histological observation

The rats were sacrificed right after hemodynamic measurements and the hearts were harvested. After trimming, hearts were fixed with 4% formaldehyde and then embedded in paraffin. The tissues were cut into 4‐μm‐thick sections that were subjected to hematoxylin and eosin staining.

### Bronchoalveolar lavage fluid acquirement and ELISA

After the animals were sacrificed, intratracheal injection of sterile PBS was performed to acquire bronchoalveolar lavage fluid (BALF). Lungs were lavaged three times with sterile PBS. Then the acquired BALF was centrifuged at 800 ***g*** at 4 °C for 10 min to separate cell pellets and supernatant. By using commercial ELISA kits, the concentrations of HMGB1 (Shino‐Test Corporation, Tokyo, Japan, no. 326054329), secretory RAGE (R&D Systems, Minneapolis, MN, USA, no. DRG00), TGFβ1 (Invitrogen/Thermo Fisher Scientific, Waltham, MA, USA, no. EHTGFBI) and IL6 (Invitrogen/Thermo Fisher Scientific, no. ECIL6) were determined. The ELISA protocols were in accordance with the manufacturers’ instructions.

### Electrophoretic mobility shift assay

The binding activity of PPARγ was assessed by electrophoretic mobility shift assay (EMSA). The oligonucleotide sequence used for peroxisome proliferator response elements was 5′‐CAAACTAGGTCAAAGGTCA‐3′. The concentration of salt was adjusted to avoid bias from the effect of salt on binding. Nuclear protein was used for the binding assay. Oligonucleotide was labeled with [γ^32^P]ATP with the T4 polynucleotide kinase kit (Promega, Madison, WI, USA, no. M4101) as per the manufacturer's instructions. The binding was implemented with EMSA/Gel‐shift Binding Buffer (Beyotime Biotechnology, Shanghai, China, no. GS006). The nuclear protein complex was separated from free probes by electrophoresis in a native polyacrylamide gel (5%) with EMSA/Gel‐shift Running Buffer (Beyotime Biotechnology, no. GS005). The specificity of binding was evaluated by addition of 100‐fold excess of unlabeled double‐stranded oligonucleotides. After separation, gel was transferred to 3MM filter paper (Whatman/GE Healthcare, Chicago, IL, USA), dried and visualized by exposure to X‐ray film at −80 °C for 20 h.

### Western blotting

Lung tissue samples were homogenized by using a radioimmunoprecipitation assay lysis buffer system (Santa Cruz Biotechnology, Dallas, TX, USA) supplemented with PMSF (Santa Cruz). After centrifugation, the nuclear protein and cytoplasmic protein were isolated by using a Nuclear and Cytoplasmic Protein Extraction Kit (Beyotime Biotechnology, no. P0027). Protein concentration was detected with an Enhanced BCA Protein Assay Kit (Beyotime Biotechnology, no. P0009). Samples were subjected to SDS/PAGE. Then the separated proteins were transferred to polyvinylidene fluoride or nitrocellulose filter membranes (EMD‐Millipore, Billerica, MA, USA). After blocking, the membranes were incubated with specific primary antibodies against PPARγ (Abcam, Cambridge, MA, USA, no. Ab45036), phosphorylated PPARγ (Abcam, no. Ab3484), HO1 (Invitrogen/Thermo Fisher Scientific, no. MA1‐112), HMGB1 (Invitrogen/Thermo Fisher Scientific, no. 10326‐H01H‐25), RAGE (Abcam, no. PA5‐48057), TGFβ1 (Sigma‐Aldrich, St. Louis, MO, USA, no. SRP3170), IL6 (Sigma‐Aldrich, no. SAB4301665), glyceraldehyde 3‐phosphate dehydrogenase (GAPDH; Abcam, no. MA5‐15738‐BTIN) or histone H1 (Abcam, no. MA5‐13750). Horseradish peroxidase‐conjugated secondary antibodies were then used to incubate the membranes. The immunoblots were visualized with SuperSignal West Pico chemiluminescent substrate (Thermo Fisher Scientific, no. 34078) on X‐ray film.

### Statistical analysis

Values in this study are presented as the mean ± SD. Data were analyzed with spss v.17.0 (SPSS Inc., Chicago, IL, USA) by using one‐way ANOVA or Student's *t* test. *P* < 0.05 was considered statistically significant.

## Results

### Baicalin administration relieved pulmonary hypertension and RV hypertrophy in infant rats with PH

**Figure 1:**
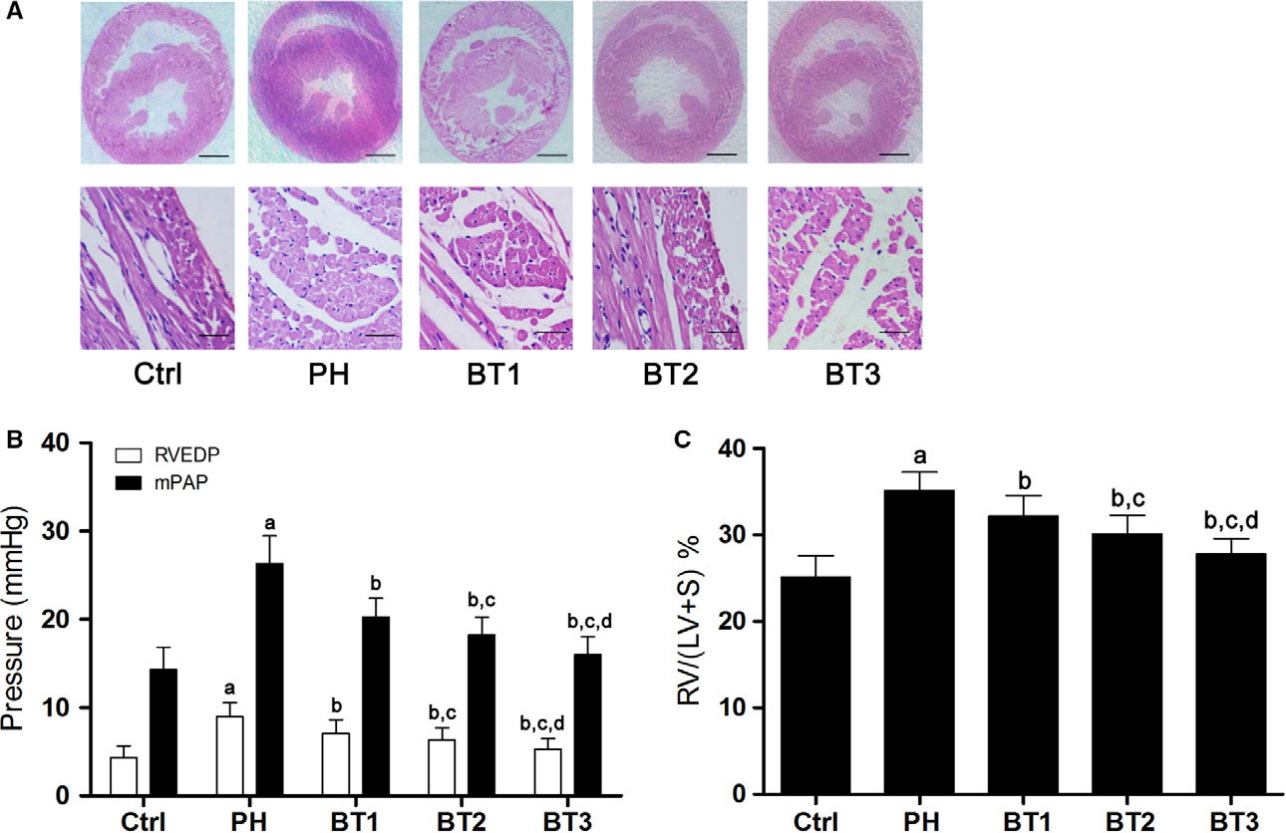
Effects of baicalin on pulmonary arterial pressure and RV structure and function in infant rats with PH. (A) Captured images of hematoxylin and eosin stained heart tissue harvested from the Ctrl, PH, BT1, BT2 and BT3 groups shown at different magnifications. Scale bars in each image stands for 1 mm or 50 μm at different magnifications. (B) The RVEDP (white columns) and mPAP (black columns) in infant rats with PH in the Ctrl, PH, BT1, BT2 and BT3 groups. (C) The ratio of the mass of right ventricle to left ventricle plus septum (RV/LV+S) in Ctrl, PH, BT1, BT2 and BT3 groups. a–d: differences were significant when compared with Ctrl (a), PH (b), BT1 (c) and BT2 (d).

As demonstrated in Fig. 1, according to the histological examination, the size and hypertrophy of the right ventricle were increased in PH, and this was attenuated by baicalin in a dosage‐dependent manner. Hypoxia induction increased mPAP, RVEDP and the RV/(LV+S) ratio in infant rats, and this was significantly attenuated by baicalin in a dosage‐dependent manner.

### Baicalin attenuated airway inflammation by suppressing HMGB1/RAGE in infant rats with PH

**Figure 2:**
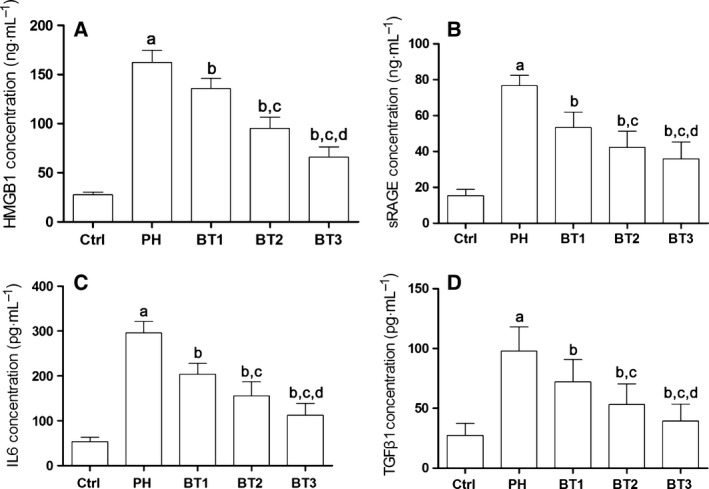
Effects of baicalin on airway inflammation in infant rats with PH. (A) HMGB1 concentrations in BALF harvested from the Ctrl, PH, BT1, BT2 and BT3 groups. (B) secretory RAGE concentrations in BALF harvested from the Ctrl, PH, BT1, BT2 and BT3 groups. (C) IL6 concentration in BALF harvested from the Ctrl, PH, BT1, BT2 and BT3 groups. (D) TGFβ1 concentrations in harvested BALF in the Ctrl, PH, BT1, BT2 and BT3 groups. a–d: differences were significant when compared with Ctrl (a), PH (b), BT1 (c) and BT2 (d).

BALF was harvested immediately after the animals were sacrificed. As shown in Fig. 2, we found that the content of TGFβ1, IL6, HMGB1 and RAGE increased significantly in BALF from infant rats with PH. However, the treatment of baicalin dramatically attenuated the incremental content of TGFβ1, IL6, HMGB1 and RAGE in infant rats with PH in a dosage‐dependent manner.

### Baicalin administration up‐regulated PPARγ activity in infant rats with PH

**Figure 3:**
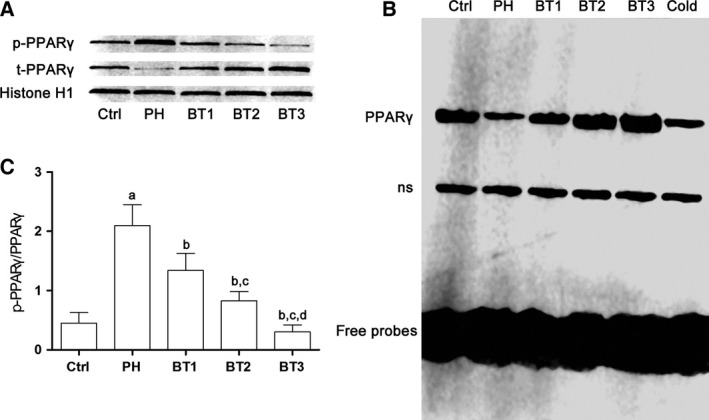
Effects of baicalin on PPARγ activation in infant rats with PH. (A) Top: immunoblots of phosphorylated PPARγ (p‐PPARγ), total PPARγ (t‐PPARγ) and histone H1 in nuclear extracts of lung tissue harvested from infant rats with PH. Botttom: the ratio of p‐PPARγ to t‐PPARγ indicating the phosphorylation of PPARγ in lung tissue harvested from the Ctrl, PH, BT1, BT2 and BT3 groups. a–d: differences were significant when compared with Ctrl (a), PH (b), BT1 (c) and BT2 (d). (B) EMSA results. Lanes 1–5: probed nuclear protein samples from Ctrl, PH, BT1, BT2 and BT3. Lane 6: competition assay using a 100‐fold excess of unlabeled peroxisome proliferator response element oligonecleotide. ns, non‐specific bands.

According to a previous study, phosphorylated PPARγ (phospho‐PPARγ) is an inhibitor of PPARγ transcriptional activity 19. As demonstrated in Fig. 3, we found that the expression level of total PPARγ decreased while the phospho‐PPARγ level increased significantly, leading to a dramatic increase of the phospho‐PPARγ/total PPARγ ratio in lungs from infant rats with PH. EMSA results also indicated the deactivation of PPARγ, which was evidenced by the down‐regulation of PPARγ DNA‐binding ability. However, in lungs from infant rats with PH treated with baicalin, the phospho‐PPARγ/total PPARγ ratio as well as the DNA‐binding activity of PPARγ recovered significantly in a dosage‐dependent manner.

### Baicalin treatment attenuated airway inflammation through activating PPARγ/HO1, which reduced HMGB1/RAGE level

**Figure 4:**
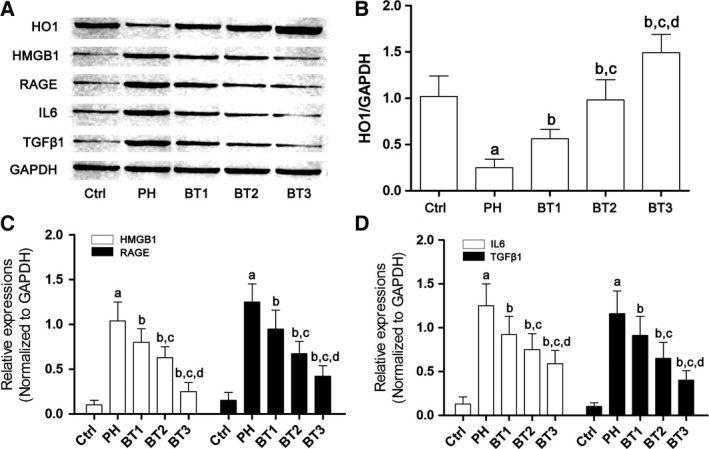
Effects of baicalin on the HO1–HMGB1–RAGE pathway in infant rats with PH. (A) Immunoblots of HO1, HMGB1, RAGE, IL6, TGFβ1 and GAPDH in lung tissue harvested from infant rats with PH. (B) The relative expression level of HO1 in the Ctrl, PH, BT1, BT2 and BT3 groups. (C) The relative expression of HMGB1 (white columns) and RAGE (black columns) in the Ctrl, PH, BT1, BT2 and BT3 groups. (D) The relative expression of IL6 (white columns) and TGFβ1 (black columns) in the Ctrl, PH, BT1, BT2 and BT3 groups. a–d: differences were significant when compared with Ctrl (a), PH (b), BT1 (c) and BT2 (d).

As shown in Fig. 4, in lung tissue from infant rats with PH, the expression level of HO1 was significantly decreased and HMGB1/RAGE signaling was activated. As a result, the expression levels of inflammatory cytokines, namely TGFβ1 and IL6, increased dramatically. However, the administration of baicalin impaired activation of this inflammatory signaling. We found that baicalin treatment inhibited the activation of HMGB1/RAGE signaling by increasing HO1 expression. As a result, the expression levels of IL6 were reduced in lung tissue from infant rats with PH.

## Discussion

Though relatively rare, PH plays a role in morbidity and even mortality, which is characterized by heart failure and premature death 20. Inflammation has been strongly suggested to play an important role in initiation and development of PH. IL6 is recognized as one of the reactive proinflammatory cytokines in responses to various pathogens. Previous studies indicated that increased IL6 was one of the risk factors for PH patients 21. IL6 level was also reported to be correlated with symptoms and prognosis of patients with PH 22, 23. Activation of IL6 would lead to the initiation of expression of TGFβ1, which participates in and promotes pulmonary vascular remodeling. It was reported that augmented TGFβ1 was associated with increased reticular basement membrane thickness in airway mucosa 24. As one of the downstream effecters of TGFβ1, CTGF could further promote the production of collagen and fibronectin, which are critical for airway remodeling 25. In the current study, hypoxia was used to induce PH in infant rats. Hemodynamic evaluation showed that mPAP and RVEDP were increased in PH animals, which also exhibited RV hypertrophy. Furthermore, the contents of IL6 and TGFβ1 were found to be increased in both BALF and lung tissue of infant rats with PH.

Herbal agents have been attracting researchers’ attention because of their wide spectrum of biological activities. Baicalin is a flavonoid extracted from *S. baicalensis* Georgi, which is called huang qin in traditional Chinese medicine. Baicalin has been shown to exhibit various pharmacological activities including antioxidation, antiproliferation, antitumor and anti‐inflammation effects. Several studies have recently demonstrated that baicalin showed curative effects in PH animal models 16, 26. In this study, we investigated the effects of baicalin on infant rats with PH. After administration of baicalin, the mPAP was lowered. As a result, both the RVEDP and RV hypertrophy were dramatically attenuated. We further explored the potential mechanisms. The results showed that the production of HMGB1, RAGE, and IL6 and TGFβ1 were reduced in BALF and lung tissue in infant rats with PH that received baicalin treatment. These results suggested that HMGB1/RAGE signaling might be the molecular target for baicalin.

In lung tissue from the infant animal PH model, compared with normal control animals, the expression of phospho‐PPARγ, which is an inhibitor of PPARγ signaling, was intensively increased while the PPARγ DNA‐binding activity was critically reduced. The result suggested that the deactivation of PPARγ signaling participates in PH. We found that the expression of HO1, the downstream effector of PPARγ, was significantly down‐regulated in animals with PH. It was reported that the induction of HO1 led to the anti‐inflammatory effects of PPARγ activation 27. An investigation suggested that HO1 conducted the signal from PPARγ activation to inhibition of the HMBG1–RAGE axis, which was believed to initiate transcription of proinflammatory factors 12. Similar results were observed in infant rats with PH in this study. The HMGB1–RAGE axis was found to be activated in infant rats with PH. According to these results, we concluded that PPARγ/HO1 signaling was deactivated in infant rats with PH. As a result, the inhibitory effect of HO1 on the HMGB1–RAGE axis was impaired. Activated HMGB1 enhanced the production of IL6 and TGFβ1, which contributed to pulmonary vascular remodeling through RAGE.

Several flavonoids including chrysin, kaempferol and apigenin could activate PPARγ signaling by direct interaction. Investigations showed that the unique molecular structures of these flavonoids were crucial for the interaction. For instance, the 4′ position of the B ring, 5′ and 7′ positions of the A ring and the C2–C3 double bond of the C ring were the essential structures for activation of PPARγ signaling 28. As one of the flavonoids, baicalin also showed activity as a PPARγ signaling activator 29. Indeed, in this study, we found baicalin to be an activator of PPARγ in infant rats with PH. The administration of baicalin significantly promoted the PPARγ activity by suppressing expression of phospho‐PPARγ and increasing PPARγ DNA‐binding activity in infant rats with PH. Consequently, the expression of its downstream mediator HO1 was elevated by baicalin treatment. For this reason, the activation of the HMBG1–RAGE axis was suppressed and the production of IL6 and TGFβ1 was also reduced. Eventually, according to our observation, the mPAP, RV function and RV hypertrophy were improved in infant rats with PH treated with baicalin.

In summary, our study demonstrated that the deactivation of PPARγ signaling is one of the mechanisms of PH. Consequently, as the downstream mediator of PPARγ, HO1's expression was down‐regulated. As a result, the HMGB1–RAGE axis was activated to induce airway inflammation and pulmonary vascular remodeling. Baicalin showed activity as an activator of PPARγ, which attenuated PH in infant rats. This curative effect was coupled with HO1 induction and HMGB1–RAGE axis suppression. It can be assumed from this study that activating PPARγ would be an effective strategy in treating PH. Moreover, baicalin and baicalin‐containing compounds may be of therapeutic value in PH.

## Author contributions

ZC performed the experiments and wrote the manuscript. QW designed the experiments and performed the statistical analysis.
